# Expression ratio of the TGFβ-inducible gene *MYO10* is prognostic for overall survival of squamous cell lung cancer patients and predicts chemotherapy response

**DOI:** 10.1038/s41598-018-27912-1

**Published:** 2018-06-22

**Authors:** D. Dvornikov, M. A. Schneider, S. Ohse, M. Szczygieł, I. Titkova, M. Rosenblatt, T. Muley, A. Warth, F. J. Herth, H. Dienemann, M. Thomas, J. Timmer, M. Schilling, H. Busch, M. Boerries, M. Meister, U. Klingmüller

**Affiliations:** 1 Division Systems Biology of Signal Transduction, German Cancer Research Center (DKFZ), 69120 Heidelberg, Germany; 2 Translational Lung Research Center Heidelberg (TLRC), German Center for Lung Research (DZL), 69120 Heidelberg, Germany; 3 Faculty of Biosciences, Heidelberg University, 69120 Heidelberg, Germany; 4 Translational Research Unit, Thoraxklinik at University Hospital Heidelberg, 69126 Heidelberg, Germany; 5 Institute of Molecular Medicine and Cell Research, University of Freiburg, 79104 Freiburg, Germany; 6 Institute of Physics, University of Freiburg, 79104 Freiburg, Germany; 7 Institute of Pathology, Heidelberg University, 69120 Heidelberg, Germany; 8 Department of Pneumology and Critical Care Medicine, Thoraxklinik at University Hospital Heidelberg, 69126 Heidelberg, Germany; 9 Department of Surgery, Thoraxklinik at University Hospital Heidelberg, 69126 Heidelberg, Germany; 10 Department of Thoracic Oncology, Thoraxklinik at University Hospital Heidelberg, 69126 Heidelberg, Germany; 11 Freiburg Centre for Systems Biology, University of Freiburg, 79104 Freiburg, Germany; 12 BIOSS Centre for Biological Signaling Studies, University of Freiburg, 79104 Freiburg, Germany; 13 Institute of Experimental Dermatology & Institute of Cardiogenetics, University of Lübeck, 23562 Lübeck, Germany; 14 German Cancer Consortium (DKTK), German Cancer Research Center (DKFZ), 69120 Heidelberg, Germany

## Abstract

In lung cancer a deregulation of Transforming Growth Factor-β (TGFβ) signaling has been observed. Yet, the impact of TGFβ in squamous cell carcinoma of the lung (LUSC) remained to be determined. We combined phenotypic and transcriptome-wide studies and showed that the stimulation of the LUSC cell line SK-MES1 with TGFβ results in an increase of migratory invasive properties. The analysis of the dynamics of gene expression by next-generation sequencing revealed that TGFβ stimulation orchestrates the upregulation of numerous motility- and actin cytoskeleton-related genes. Among these the non-muscle myosin 10 (*MYO10*) showed the highest upregulation in a LUSC patient cohort of the Cancer Genome Atlas (TCGA). Knockdown of *MYO10* abrogated TGFβ-induced collagen gel invasion of SK-MES1 cells. The analysis of *MYO10* mRNA expression in paired tissues of 151 LUSC patients with corresponding 80-month clinical follow-up data showed that the mRNA expression ratio of *MYO10* in tumor and tumor-free tissue is prognostic for overall survival of LUSC patients and predictive for the response of these patients to adjuvant chemotherapy. Thus, *MYO10* represents a new clinical biomarker for this aggressive disease and due to its role in cellular motility and invasion could serve as a potential molecular target for therapeutic interventions in patients with LUSC.

## Introduction

Non-small-cell lung cancer (NSCLC) is the leading cause of cancer-related mortalities. Early metastasis and therapy resistance are the main features that result in high mortality among lung cancer patients^1^. Adenocarcinoma of the lung (LUAD) and squamous cell carcinoma of the lung (LUSC) are the two major subtypes of NSCLC. Although the prevalence of LUSC in developed countries is declining, it still accounts for about 25% of NSCLC cases^2^. Despite the great progress in developing targeted approaches in LUAD, therapeutic options for LUSC remain very limited as driver oncogene mutations are uncommon^3^. For decades platinum-based chemotherapy has been the gold standard for first-line therapy for LUSC patients. However, in a significant proportion of patients cancer cells are resistant to chemotherapy and the disease rapidly progresses^4^. Thus, there is an urgent need to gain insights into mechanism contributing to LUSC in order to establish mechanism-based biomarkers that help clinicians to identify patients at the highest risk for disease progression and therapy resistance.

Both early metastasis and therapy resistance are attributed to cancer cells undergoing epithelial-to-mesenchymal transition (EMT) and acquiring a more invasive phenotype with cancer stem cell-like properties^5^. Tumor cells harboring EMT features were repeatedly reported to localize at the invasive front of the tumor, hence mediating cancer cell dissemination and metastasis^6^.

There is growing evidence that deregulated TGFβ signaling contributes to the acquisition of an EMT phenotype by lung cancer cells. In the context of LUSC, elevated TGFβ1 levels were correlated with poor patient prognosis^7^ and over-activation of the TGFβ pathway was reported as a common feature in lung cancer^8^. Moreover, the EMT phenotype was widely observed in surgically resected specimens and associated with a worse clinical outcome and chemoresistance^9^. However, a mechanistic understanding of TGFβ-induced changes and their impact on LUSC progression remained to be established. Therefore, we combined phenotypic and transcriptome-wide approaches to determine TGFβ-induced dynamic changes in the transcriptome of a LUSC cell line and thereby derived a candidate prognostic biomarker that we validated in a clinical cohort.

## Results

### TGFβ treatment enhances pro-tumorigenic properties of LUSC cells

**Figure 1:**
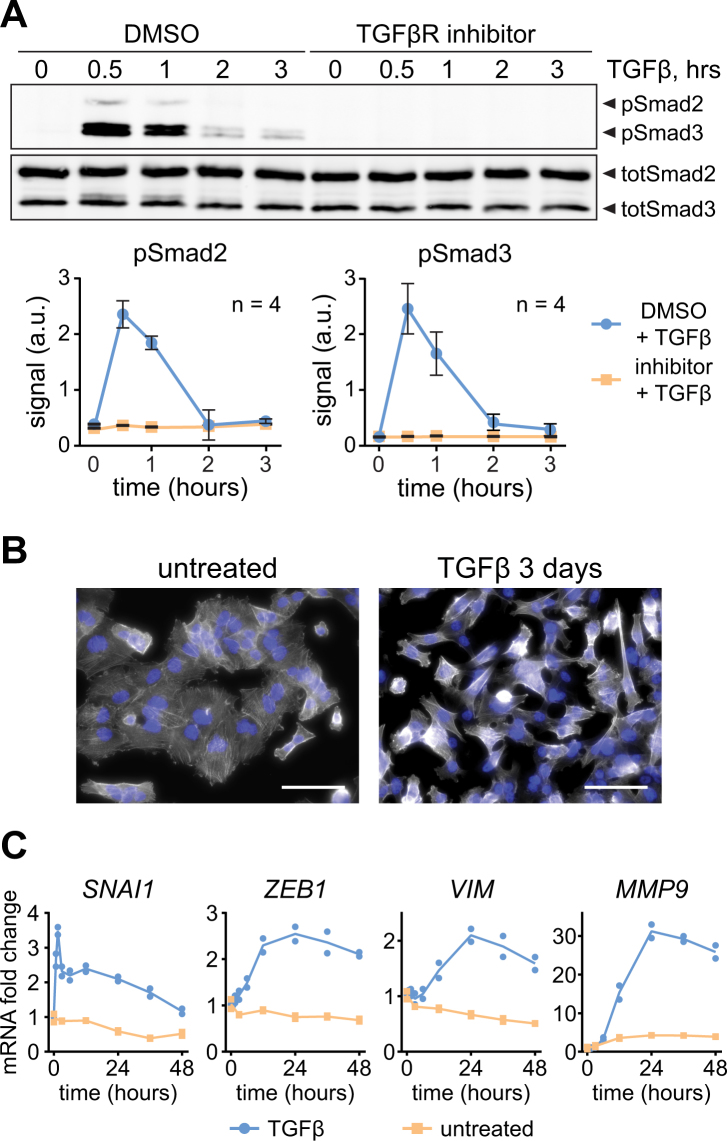
TGFβ treatment triggers EMT in SK-MES1 cells. (**A**) TGFβ induces Smad2/3 phosphorylation in TGFβR1-dependent way. SK-MES1 cells were pretreated with TGFβR1 inhibitor SB-431542 or DMSO and then stimulated with 2 ng/ml TGFβ1. Data presented correspond to mean and SD, n is the number of independent experiments. Additional replicates are shown in Supplementary Fig. S1A. Full-length blots are shown in Supplementary Fig. S6. (**B**) Prolonged exposure of SK-MES1 cells to TGFβ1 induces acquisition of EMT-like morphology. Cells were either stimulated with 2 ng/ml TGFβ1 or left untreated for 3 days, fixed and stained for F-actin (white) and DNA (blue). Scale bar corresponds to 50 µm. (**C**) EMT marker genes are upregulated upon TGFβ1 treatment. Growth factor-depleted SK-MES1 cells were stimulated with 2 ng/ml TGFβ1 or left untreated. RNA was extracted and analyzed using qRT-PCR. mRNA expression was normalized to four housekeepers: *GUSB*, *HPRT*, *GAPDH* and *G6PD*. Each dot represents a biological replicate. A second independent experiment is shown in Supplementary Fig. S1B.

To study the impact of TGFβ on LUSC cells, we used the LUSC cell line SK-MES1 as a cellular model system. By quantitative immunoblotting we showed that TGFβ-induced phosphorylation of Smad2 and Smad3 in SK-MES1 cells reached a maximum after 30 min and declined thereafter (Fig. 1A and Supplementary Fig. S1A). SK-MES1 cells usually grow in tight epithelial colonies, but after treatment with TGFβ they lost cell-cell contacts and acquired an elongated spindle-shaped morphology (Fig. 1B), a feature commonly observed upon TGFβ-induced epithelial-to-mesenchymal transition (EMT). In line with these morphological alterations, TGFβ treatment of SK-MES1 cells induced the mRNA expression of classical EMT markers such as *SNAI1*, *ZEB1*, *VIM* and *MMP9* (Fig. 1C and Supplementary Fig. 1B).

**Figure 2:**
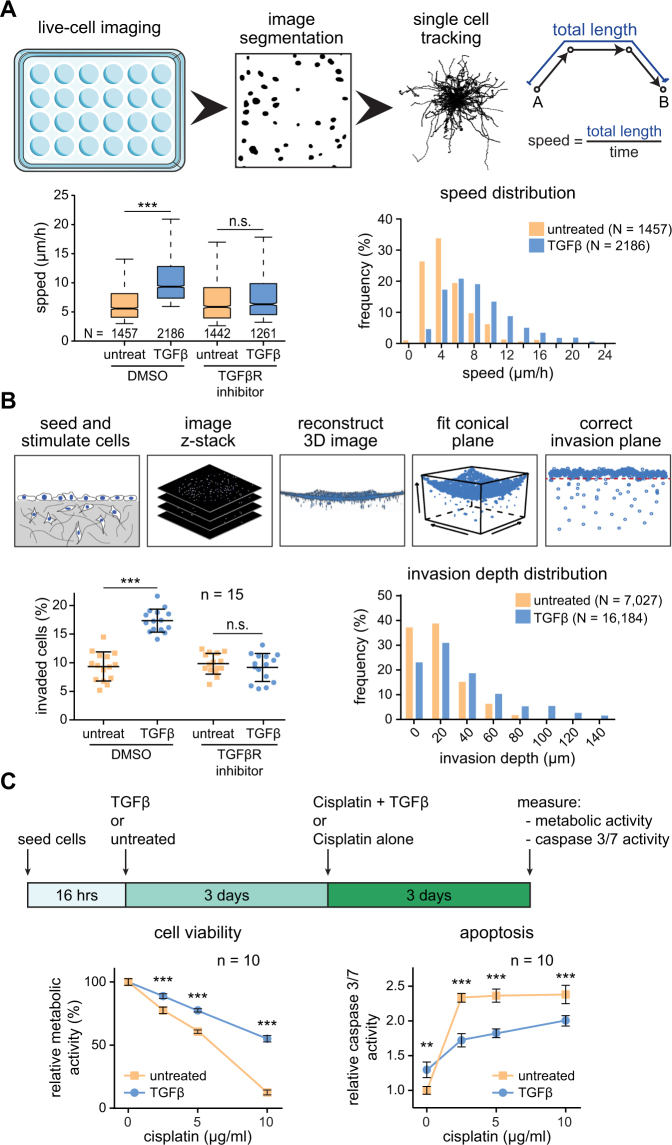
TGFβ treatment increases invasiveness and cisplatin resistance of squamous lung carcinoma cells SK-MES1. (**A**) Top, schematics of the 2D migration assay. Bottom, migratory properties of SK-MES1 cells are increased upon TGFβ treatment. Cells were seeded in 24-well plate, pretreated with either SB-431542 or DMSO, stimulated with 2 ng/ml TGFβ1 and imaged for 60 hours. Migration speed from each single cell track was quantified. Center lines show the medians; box limits indicate the 25th and 75th percentiles; whiskers extend to 5th and 95th percentiles, N indicates the number of quantified single cell tracks per condition. Additional independent experiments are shown in Supplementary Fig. S2A. Statistical analysis was performed using one-way ANOVA; ***P < 0.001; n.s., not significant. (**B**) Top, schematics of the collagen 3D invasion assay. Bottom, TGFβ stimulation increases number of invading cells and the average invasion depth. SK-MES1 cells were seeded in 96-well plates with precast collagen gels, allowed to attach overnight, growth factor-depleted for three hours, pretreated with either SB-431542 or DMSO, stimulated with 2 ng/ml TGFβ1, allowed to invade for four days, stained with Hoechst and imaged with a confocal microscope. The number of invaded cells and invasion depth were assessed. One representative experiment is shown. Data are presented as median and SD, every dot corresponds to a biological replicate (n = 15). N indicates the number of invaded cells. Additional independent experiments are shown in Supplementary Fig. S2B. Statistical analysis was performed using one-way ANOVA; ***P < 0.001; n.s., not significant. (**C**) Top, schematics of the experimental setup. Bottom, pre-treatment with TGFβ1 reduces sensitivity of SK-MES1 cells to cisplatin treatment. Cells were seeded in 96-well plate, stimulated with either 2 ng/ml TGFβ1 or left untreated for 3 days and then exposed to increasing doses of cisplatin for 3 days. Cell viability and caspase 3/7 activity were assessed. Data presented correspond to mean and SD, n is the number of biological replicates. Additional independent experiments are shown in Supplementary Fig. S1C. Statistical analysis was performed using one-way ANOVA; **P < 0.01; ***P < 0.001.

We explored how the activation of TGFβ signal transduction and target gene expression as well as the morphological changes translated into altered phenotypic responses. To this aim, we established workflows to quantitatively assess at the single cell level the impact of TGFβ on SK-MES1 cells in a 2D cell migration assay and a 3D collagen invasion assay (Fig. 2A,B upper panels). This included the development of a mathematical algorithm to correct for the concave surface of the collagen gels to improve the reliability of quantifications in the 3D collagen invasion assay. In the 2D migration assay we observed by analyzing more than 1000 of single cell tracks per condition that the TGFβ treatment resulted in a two-fold increase in migration speed (from 4 to 8 µm/h) (Fig. 2A and Supplementary Fig. S2A). Co-treatment with a type I TGFβ receptor inhibitor prevented this effect. In the 3D collagen invasion assay TGFβ treatment resulted in a two-fold increase in the number of collagen-invaded SK-MES1 cells. Some of the TGFβ-treated SK-MES1 cells invaded more than 100 µm into the dense collagen gels, while untreated cells invaded on average not more than 20 µm (Fig. 2B and Supplementary Fig. S2B). The increase in the invasion capacity was TGFβ-specific because it was abolished by co-treatment with a type I TGFβ receptor inhibitor.

It was reported that the EMT phenotype correlates with increased resistance to chemotherapy^10^. To examine the impact of TGFβ on the resistance of SK-MES1 cells to cisplatin, a cell viability assay based on metabolic activity and an apoptosis assay based on caspase 3/7 activity was employed (Fig. 2C). We observed that pre-treatment of SK-MES1 with TGFβ for 3 days resulted in a 4.4-fold increase of viable cells after 3 days exposure to 10 µg/ml cisplatin (Fig. 2C and Supplementary Fig. S2C). Likewise, pretreatment with TGFβ reduced the caspase 3/7 activity across all tested doses of cisplatin by 25% (Fig. 2C and Supplementary Fig. S2D). Collectively, these data indicate that SK-MES1 cells acquire a more aggressive phenotype upon exposure to TGFβ.

### Multiple actin cytoskeleton- and motility related genes are upregulated in LUSC cells upon TGFβ stimulation

**Figure 3:**
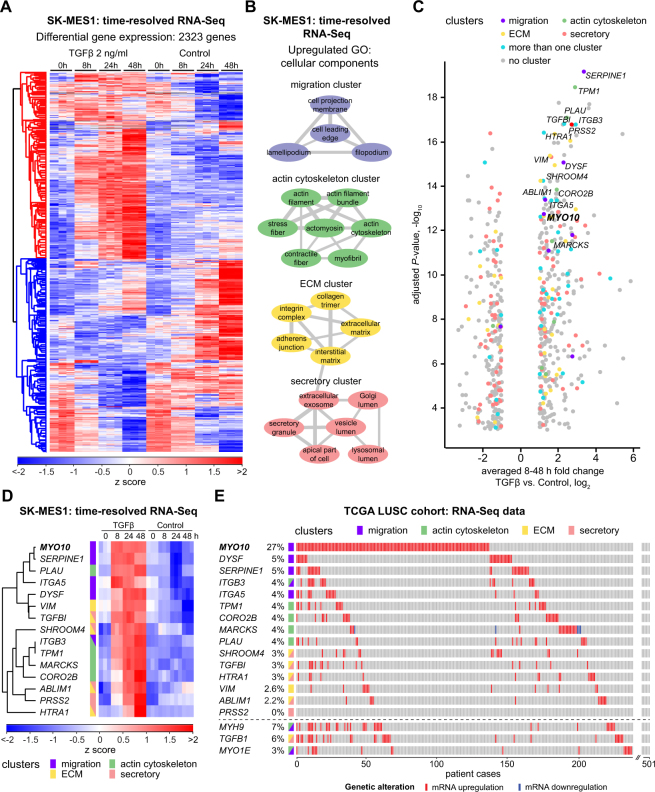
TGFβ treatment оf LUSC cells results in upregulation of migration- and actin cytoskeleton-related genes. (**A**) Non-supervised hierarchical clustering of z-scored differentially regulated 2323 genes (adjusted *P*-value < 0.01) between TGFβ-treated and untreated conditions. SK-MES1 cells were stimulated with 2 ng/ml TGFβ or left untreated. RNA was extracted and sequenced using HiSeq 4000. (**B**) Clusters of significantly upregulated GO cellular component gene sets between TGFβ-treated and untreated conditions. Significantly upregulated GO terms (adjusted *P*-value < 0.01) were visualized using REVIGO (allowed similarity 0.5). Thickness of connecting grey lines corresponds to the similarity of the GO terms. Only clusters that consist of at least two GO terms are displayed. (**C**) Volcano plot of differentially regulated genes between TGFβ-treated and untreated conditions. Fold change of averaged 8–48 h time points between both conditions is displayed. Only significantly regulated genes (adjusted *P*-value < 0.01) with a fold change of at least two are shown. Five most regulated genes from each cluster of upregulated GO cellular component gene sets are indicated with corresponding colors. Grey circles indicate differentially regulated genes that do not belong to any of the four clusters. (**D**) Time-resolved dynamics of top differentially regulated candidate genes from each of the clusters. Top five genes from each of the four clusters with the lowest adjusted *P*-values and fold change of at least two after normalization to untreated samples were selected as candidates. In case the same gene belonged to different clusters and satisfied the inclusion criteria, it was marked as belonging to both clusters. Single gene plots are shown in the Supplementary Fig. S3A. (**E**) TCGA LUSC cohort RNA-Seq expression data of selected candidate genes sorted by frequency of mRNA upregulation. *MYH9*, *TGFB1* and *MYO1E* genes were additionally included.

To elucidate mechanisms that contribute to TGFβ-induced cancer cell invasion and increased chemotherapy resistance in LUSC cell line SK-MES1, we performed a time-resolved whole-transcriptome RNA-Seq analysis of SK-MES1 cells that were treated with TGFβ for up to 48 hours or were left untreated. Genes were considered as differentially regulated if their overall mRNA expression dynamics in treated versus untreated cells was significantly different (multiple testing adjusted *P*-value < 0.01). In total expression of 2323 genes significantly changed in response to TGFβ treatment (Fig. 3A). The resulting list of differentially regulated genes was used for Gene Set Enrichment Analysis (GSEA) to identify regulated gene ontology (GO) terms of cellular components, which were subsequently visualized with the REVIGO tool^11^ to establish clusters with distinct gene expression patterns. This approach revealed a preferential regulation of four gene clusters encoding actin cytoskeleton-, motility-, ECM- and secretory-related proteins (Fig. 3B). To narrow down the list of potential candidates involved in mediating the TGFβ-induced invasive properties of LUSC cells, the five genes per cluster with the lowest multiple testing-adjusted *P*-values and with at least two-fold upregulation after normalization to untreated samples were selected. Because some of the genes were among the top five candidates in more than one cluster this resulted in a list of 15 TGFβ-regulated genes (Fig. 3C,D and Supplementary Fig. S3A). Interestingly, genes identified as candidates in our approach included *MYO10, SERPINE1, ITGB3, ITGA5, TGFBI, VIM* and *MARCKS*. These TGFβ-regulated genes were previously associated with increased cancer invasiveness, chemoresistance and worse clinical outcome in different cancer entities including breast, lung cancer (both LUSC and LUAD), invasive melanoma and prostate cancers^12–17^. To determine which of these TGFβ-regulated genes are relevant in the context of LUSC, we evaluated the alterations of mRNA levels of the selected 15 candidate genes in a cohort of 501 LUSC patients from the Cancer Genome Atlas (TCGA) (Fig. 3E). Strikingly, of those genes the *MYO10* gene was the top candidate as it was upregulated in 27% of the patients, whereas an upregulation of the mRNAs of the other genes was only observed in 2–5% of the LUSC patients. Interestingly, the genes with the highest percentage of mRNA upregulation in LUSC patients belonged either to the migration or the actin cytoskeleton clusters, while genes from the ECM and secretory clusters were rarely altered in LUSC patients, although several of the genes from these clusters showed a high fold increase in SK-MES1 cells upon TGFβ treatment (Fig. 3C,D and Supplementary Fig. S3A). Given the prominent upregulation of *MYO10* expression in LUSC patients and the pivotal role of non-muscle myosins in mediating cancer cell invasion in multiple cancer entities^18^, we examined whether other myosin-encoding genes scored high in our analysis but were not among the top five TGFβ-regulated genes. Indeed, in the LUSC patients of the TCGA cohort the second and third most regulated genes encoding myosins were *MYH9* and *MYO1E*, which were previously implicated in cancer progression^19,20^. Both of these myosin genes were upregulated in LUSC patients of the TCGA cohort with *MYH9* being overexpressed in 7% of the cases (Fig. 3E lower panel). A significant co-occurrence of an upregulation of the mRNAs of *MYO10*, *MYH9*, *MYO1E* and *TGFB1* was observed in the LUSC patients of the TCGA cohort (Supplementary Fig. S3B), suggesting that the exposure of tumor cells to elevated levels of TGFβ might have stimulated upregulation of motility and invasion-related myosins. Therefore, all three myosin genes were included for further analysis.

### TGFβ-induced myosin motors are essential for TGFβ-mediated cancer cell invasion

**Figure 4:**
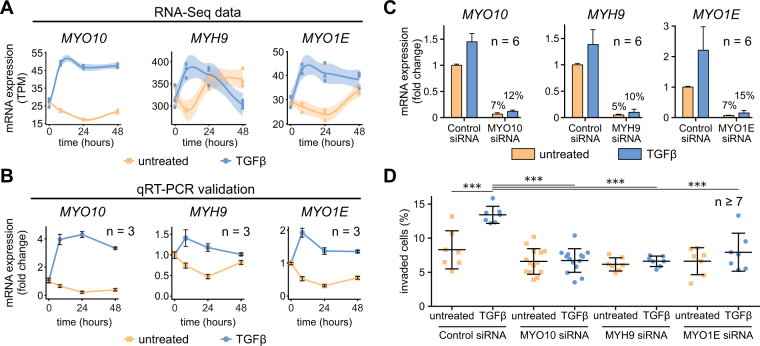
TGFβ-inducible myosins are required for TGFβ-mediated cancer cell invasion. (**A**) Time-resolved RNA-Seq data of selected myosin genes upon TGFβ treatment in SK-MES1 cells. Cells were growth factor-depleted for three hours and stimulated with 2 ng/ml TGFβ1 or left untreated. mRNA was extracted and sequenced using HiSeq 4000. Data are presented in TPM (transcripts per million) values. Each dot represents a biological replicate, shaded areas correspond to standard error. (**B**) qPCR validation of RNA-Seq data. RNA of TGFβ-treated and untreated SK-MES1 cells that was used for RNA-Seq was also analyzed with qRT-PCR. Data presented correspond to mean and SD from three biological replicates. (**C**) siRNA knockdown results in 90% knockdown efficiency of TGFβ-inducible myosins. After 36 hours of siRNA transfection SK-MES1 cells were stimulated for 1 hour with 2 ng/ml TGFβ1 or left untreated. mRNA was extracted and the knockdown efficiency was analyzed using qRT-PCR. Data represent mean and SD from six biological replicates. (**D**) Myosins knockdowns inhibit TGFβ-mediated cancer cell invasion. SK-MES1 cells were transfected with siRNAs for 36 hours and stimulated with 2 ng/ml TGFβ1 for four days. Amount of invaded cells into the collagen gel was assessed. One representative experiment is shown. Every dot corresponds to a biological replicate (n ≥ 7), black line indicates the median. Additional independent experiment is shown in Supplementary Fig. S4C. Statistical analysis was performed using one-way ANOVA; ***P < 0.001.

To examine the biological importance of the candidate TGFβ-induced myosins for LUSC, we validated the time-resolved RNA-Seq data (Fig. 4A) with time-resolved examinations by qRT-PCR of TGFβ-stimulated SK-MES1 cells (Fig. 4B). mRNA expression data assessed by both methods significantly correlated with Spearman coefficients of ρ = 0.92, 0.78 and 0.54 for *MYO10*, *MYO1E* and *MYH9* mRNAs, respectively (Supplementary Fig. S4A). All three candidate genes demonstrated strong mRNA induction upon TGFβ treatment, with *MYO10* expression being the most pronounced and most sustained for up to 48 hours. Interestingly, cisplatin treatment alone did not alter expression of *MYO10*, *MYH9* and *MYO1E* mRNAs, but slightly modulated TGFβ-induced induction when applied in combination with 2 ng/ml of TGFβ (Supplementary Fig. S4B).

Given the role of non-muscle myosins in cancer metastasis, we studied the effect of gene silencing on the ability of the SK-MES1 cells to invade 3D collagen gels in response to TGFβ stimulation. We used siRNA to knockdown *MYO10*, *MYH9* or *MYO1E* and achieved for all of them a knockdown efficiency of more than 85% at the mRNA level (Fig. 4C). Whereas TGFβ treatment of SK-MES1 cells transfected with control non-targeting siRNA resulted in a two-fold increase in the number of invaded cells compared to unstimulated cells (Fig. 4D), the TGFβ-enhanced invasion of SK-MES1 cells was abrogated upon downregulation of the different myosins. These results indicate that the TGFβ-induced non-muscle myosins MYO10, MYH9 and MYO1E play a non-redundant and crucial role in mediating TGFβ-regulated invasiveness of the LUSC cells.

### *MYO10* mRNA overexpression is prognostic for overall survival of patients with squamous cell carcinoma

**Figure 5:**
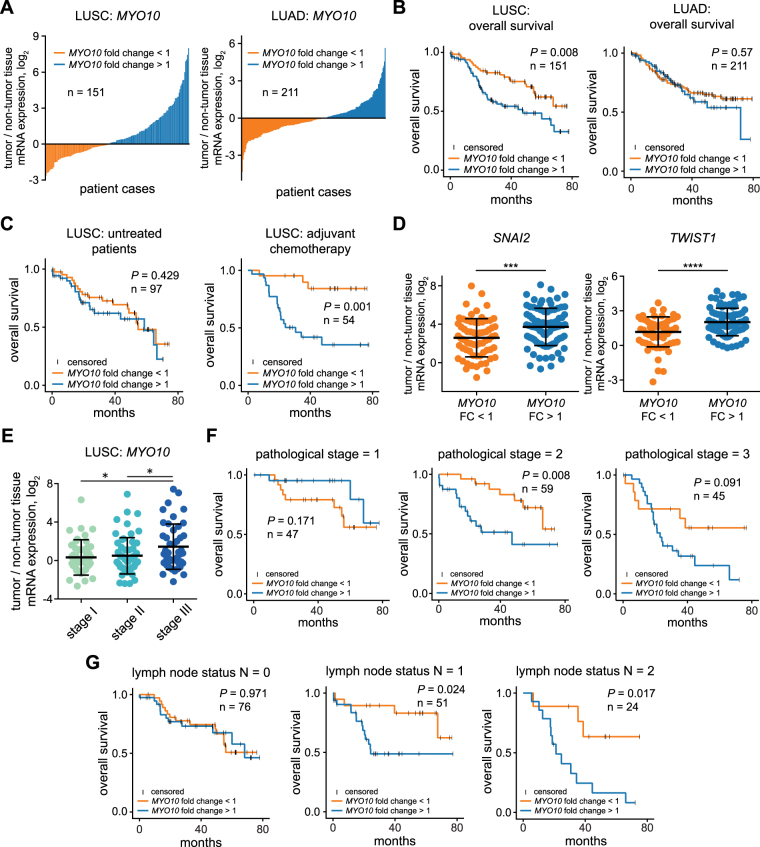
*MYO10* mRNA expression ratio is prognostic for overall survival of LUSC but not LUAD patients. (**A**) *MYO10* mRNA expression ratio of tumor and adjacent non-tumor tissues in LUSC and LUAD patients. RNA was isolated from fresh-frozen resected tissues and *MYO10* expression was measured using qRT-PCR. (**B**) Kaplan-Meier curves for overall survival using *MYO10* mRNA expression ratio in LUSC and LUAD cohorts. Significance of difference between the two groups was tested using non-parametric Mann-Whitney U test. (**C**) Kaplan-Meier curves for adjuvant chemotherapy response in *MYO10* low (left) and *MYO10* high (right) patients. Significance of difference between the two groups was tested using non-parametric Mann-Whitney U test. (**D**) Differences of *SNAI2* and *TWIST1* expression depending on *MYO10* mRNA expression ratio were tested by unpaired *t*-tests; ***P < 0.001; ****P < 0.0001. (**E**) *MYO10* expression ratio in different stages of LUSC. Differences were tested by one-way ANOVA; *P ≤ 0.05. (**F**) Kaplan-Meier curve for the pathological stages of LUSC patients. Significance of difference between the two groups was tested using non-parametric Mann-Whitney U test. (**G**) Kaplan-Meier curves for lymph node status using *MYO10* mRNA expression ratio. Significance of difference between the two groups was tested using non-parametric Mann-Whitney U test.

Actin-based protrusions and TGFβ-induced myosins are crucial for multiple phases of the metastatic cascade^18^. Among the identified TGFβ-regulated non-muscle myosins *MYO10* showed the strongest upregulation in response to TGFβ stimulation in SK-MES1 cells and the highest mRNA overexpression in LUSC patients of the TCGA cohort. Therefore, we further assessed its clinical relevance in paired tumor and tumor-free tissue from our NSCLC cohort consisting of both LUAD and LUSC patients with a similar tumor stage distribution among LUSC patients as in the TCGA LUSC cohort (Supplementary Table S1 and Supplementary Fig. S5B). For each tumor entity, patients were divided into two subgroups, *MYO10* fold change <1 and *MYO10* fold change >1, based on the expression ratio of *MYO10* mRNA in tumor versus tumor-free tissue (Fig. 5A). To investigate the prognostic value of the *MYO10* mRNA expression ratio, we performed Cox regression analysis (Table 1). Univariate analysis indicated that a high *MYO10* mRNA expression ratio (*P* = 0.018), gender (*P* = 0.04) as well as the pathological stages (*P* = 0.005 for pstage II and *P* < 0.001 for pstage III) were prognostic factors for the overall patient survival. The multivariate analysis suggested that a high *MYO10* mRNA expression ratio was only prognostic for LUSC patients, but not for LUAD patients (Table 2). Using the *MYO10* mRNA expression ratio to separate the patient groups, we confirmed that LUSC patients with high *MYO10* mRNA expression ratio demonstrate reduced overall survival independent of the tumor stage and treatment regimen (*P* = 0.008, Fig. 5B left), which was not observed in LUAD patients (*P* = 0.57, Fig. 5B right). Next, the LUSC patients were subdivided according to those that had no further treatment after the resection (mainly stage IB and IIA patients with a median disease-free survival of 28.4 months) and those who received adjuvant chemotherapy (mainly stage IIB and IIIA patients with a median disease-free survival of 35.6 months) (Fig. 5C and Supplementary Fig. S5C,D). This analysis showed that for untreated patients *MYO10* mRNA expression ratio expression was not predictive for overall survival (*P* = 0.429) (Fig. 5C, left panel). On the contrary, patients with low *MYO10* mRNA expression ratio strongly benefited from the adjuvant chemotherapy treatment in comparison to patients with a high *MYO10* mRNA expression ratio (*P* = 0.001) (Fig. 5C, right panel). Therefore, we conclude that *MYO10* mRNA expression ratio is predictive for the outcome of adjuvant chemotherapy treatment of LUSC patients.

**Table 1:** *MYO10* mRNA expression ratio predicts outcome in lung cancer patient cohort.

Univariate analysis of overall survival		
variable	All patients, n = 362	
	HR (95% CI)	*P*-value
*MYO10* in tumor (high vs. low expression)	1.042 (0.733–1.482)	0.818
*MYO10* in non-tumor (high vs. low expression)	0.780 (0.548–1.109)	0.166
*MYO10* ratio (up- vs. down-regulation)	1.533 (1.075–2.186)	**0.018**
Gender (m vs. f)	1.555 (1.021–2.369)	**0.04**
Age	1.018 (0.999–1.037)	0.068
Histology (LUAD vs. LUSC)	0.839 (0.590–1.192)	0.327
pStage (II vs. I)	2.059 (1.238–3.425)	**0.005**
pStage (III vs. I)	3.899 (2.409–6.311)	**<0.001**
Smoking status (smoker vs. non-smoker)	1.606 (0.811–3.180)	0.081
Smoking status (ex-smoker vs. non-smoker)	1.232 (0.633–2.397)	0.539

**Table 2:** Differential expression of *MYO10* is an independent predictor of survival in squamous cell carcinoma patients.

variable	Multivariate analysis of overall survival			
	LUAD patients, n = 211		LUSC patients n = 151	
	HR (95% CI)	*P*-value	HR (95% CI)	*P*-value
*MYO10* ratio (up- vs. down-regulation)	1.008 (0.967–1.050)	0.71	1.012 (1.006–1–018)	**<0.001**
Gender (m vs. f)	1.405 (0.832–2.373)	0.204	1.750 (0.75–4.083)	0.195
Age	1.005 (0.979–1.033)	0.695	1.032 (0.999–1.066)	0.06
pStage (II vs. I)	2.372 (1.120–5.025)	**0.024**	2.135 (1.010–4.513)	**0.047**
pStage (III vs. I)	4.966 (2.382–10.353)	**<0.001**	4.613 (2.169–9.811)	**<0.001**

Because of the observed enhanced chemoresistance of LUSC cells after TGFβ treatment (Fig. 2C) and because TGFβ-induced EMT has been associated with chemotherapy resistance in patients^9,21^, we determined the expression of EMT markers in tissue of LUSC patients. Notably, patients with an elevated *MYO10* mRNA expression ratio displayed a higher expression of EMT signature genes such as *SNAI2, TWIST1* and *VIM* (Fig. 5D and Supplementary Fig. S5A). The fact that TGFβ is one of the most potent EMT-inducers^22^ and the co-occurrence of *MYO10* and *TGFB1* mRNA upregulation in a substantial proportion of LUSC patients (Supplementary Fig. S3B) suggest that activation of TGFβ signaling might trigger the observed alterations in LUSC patients. Finally, a higher *MYO10* mRNA expression ratio was observed in patients with stage III disease (Fig. 5E), making it prognostic for patients with a higher pathological stage and affected local or distant lymph nodes (Fig. 5F,G). Taken together, our studies suggest that the mRNA expression ratio of *MYO10* can be used as a new independent prognostic biomarker for survival in patients with resected LUSC.

## Discussion

We investigated TGFβ-induced changes in signal transduction, gene expression and phenotypic responses in the LUSC cell line SK-MES1 to shed light on TGFβ-induced mechanisms that might contribute to tumor progression in LUSC. Our analysis identified the TGFβ-inducible non-muscle MYO10 as an essential mediator of TGFβ-regulated cancer cell invasion. Finally, we showed that a high *MYO10* mRNA expression ratio is an independent biomarker for patients at risk for a more aggressive course of the disease.

Previous reports regarding the importance of TGFβ signaling in LUSC progression are contradictory. It was shown that elevated TGFβ1 plasma levels and upregulated downstream gene targets, such as *TWIST1* and *SNAI2*, correlated with poor patient prognosis^23,24^, while it was also reported that reduced TGFβRII immunostaining in LUSC patients is associated with a more aggressive tumor behavior and reduced patient survival^25^. Here, we observed that exposure of the LUSC cell line SK-MES1 to TGFβ triggers changes in cell morphology that were accompanied by increased chemoresistance as well as an increase in migratory and invasive properties of the cells. These observations support a pro-metastatic role of TGFβ signaling in the context of LUSC.

There is growing evidence that aberrant upregulation of actin cytoskeleton proteins plays a central role in metastatic progression in different types of cancers^26^. Particularly, non-muscle myosins are implicated in tumor progression through their roles in cell migration and invasion^18^. It was reported that TGFβ1 stimulation of the epithelial-like lung adenocarcinoma cell line H1437 triggered upregulation of cytoskeletal proteins and induced a more aggressive cell phenotype. By integration of transcriptome and methylome data it was shown that the enrichment of actin cytoskeleton proteins correlated with poor survival of lung adenocarcinoma patients^27^. Others observed that high expression of the actin-binding filopodial-crosslinking protein fascin was prognostic for an aggressive metastatic disease and for poor survival of patients with breast and esophageal squamous cell tumors^28,29^. Here, we showed that siRNA-mediated knockdown of either *MYO10*, *MYH9* or *MYO1E* was sufficient to abrogate TGFβ-induced 3D collagen invasion of the LUSC cell line SK-MES1. These observations are in agreement with previous work demonstrating that shRNA knockdown of *MYO10* in the breast cancer cell line MDA-MB-231 inhibited Matrigel invasion and *in vivo* invasion in lung colonization and mammary fat pads assays^30^. siRNA depletion of *MYH9* in the esophageal squamous cell line KYSE-510 impaired migratory and invasive abilities in gap closure and transwell assays, respectively^31^. Dominant negative inhibition of MYO1E in RSV-transformed BHK-21 cells compromised the formation of invadosomes that are involved in matrix degradation and invasion^32^. Taken together, these observations strongly support that MYO10, MYH9 and MYO1E play essential and non-redundant roles in the functionality of the invasion machinery in cancer.

Our analysis of a cohort of 362 lung cancer patients, including 151 LUSC patients, revealed that the *MYO10* mRNA expression ratio between tumor and adjacent tumor-free tissues is an independent prognostic factor that is associated with poor overall survival in patients with resected LUSC. Expression of several other myosin motor proteins was previously reported to be prognostic for overall survival in patients with several cancer types. MYH9 expression examined by immunohistochemistry was an independent prognostic factor for overall survival in patients with resected NSCLC^20^, esophagus and bladder cancers^31,33^. Investigation of gene expression signatures in basal-like breast tumors showed a correlation between *MYO1E* mRNA expression level and poor prognosis^19^. However, to date there are no studies linking *MYO10* expression to patient survival. Yet, our results show that *MYO10* mRNA expression ratio might represent a new promising prognostic factor for survival of LUSC patients, especially considering the fact that *MYO10* was upregulated at the mRNA level in almost 30% of LUSC patients in the TCGA dataset, which is to our knowledge the highest rate of *MYO10* upregulation among all the cancer entities reported in TCGA. We observed that LUSC patients with high *MYO10* mRNA expression ratio and pre-existing lymph node metastases have significantly poorer overall survival (*P* = 0.017, Fig. 5G) alongside with increased chemotherapy resistance (Fig. 5C). Similar observations that tumor cells with increased metastatic potential are more resistant to chemotherapy treatment were previously made across several cancer types, including lung and breast cancers^34,35^. This was attributed to a higher metastatic potential and a reduced sensitivity to chemotherapeutic agents of tumor cells undergoing EMT that has been associated with a decreased proliferation rate and upregulation of resistance-related genes^36^. Therefore, the determination of the *MYO10* mRNA expression ratio in resected lung tumors could be used to discriminate between LUSC patients that would benefit from platinum-based adjuvant chemotherapy or not. Additionally, the prognostic value of the *MYO10* mRNA expression ratio is to help clinicians identify patients at higher risk of chemotherapy failure and disease progression. Those patients should not only be monitored more closely, but also potentially be considered for treatment with immune checkpoint inhibitors as an emerging treatment strategy in LUSC^37^. Finally, as it appears that a large fraction of LUSC tumors rely on TGFβ signals to promote their spread and chemoresistance, we suggest that in addition to *MYO10*, several other metastasis- and resistance-related TGFβ-inducible genes that we identified in our time-resolved transcriptome-wide study, including the non-muscle myosins *MYO1E* and *MYH9*, might comprise a promising robust prognostic gene signature for patients with resected LUSC.

Concluding, non-muscle myosin MYO10 serves as a downstream effector contributing to a more aggressive clinical course through increased migratory and invasive properties of the tumors as well as enhanced chemoresistance. We showed that the *MYO10* mRNA expression ratio can be used as an independent prognostic factor for survival of patients with resected lung squamous cell carcinoma and propose that MYO10 may represent a new molecular target for therapeutic intervention.

## Materials and Methods

### Cell lines and culture conditions

The human LUSC cell line SK-MES1 was purchased from ATCC and authenticated with a multiplexed human cell line authentication test (Multiplexion). Cells were grown in culture medium: DMEM (Lonza) supplemented with 10% FCS (Gibco), 100 U/ml penicillin and 100 μg/ml streptomycin (Gibco). For TGFβ1 (R&D Systems, #240-B-010) stimulation, growth factor-depleted medium was used: DMEM without Phenol red supplemented with 1 mg/ml BSA (Sigma), 100 U/ml penicillin, 100 μg/ml streptomycin and 2 mM L-glutamine. Cells were kept in 5% CO_2_ at 37 °C and 95% relative humidity. Cells were passaged to a maximum of 25 passages and controlled for mycoplasma contamination (Multiplexion).

### Immunoblotting

To examine the activation of the TGFβ/Smad pathway, 1.4 × 10^6^ of SK-MES1 cells were plated on 6-cm dishes (TPP, #93060). The next day, medium was replaced with growth factor-depleted DMEM for three hours, followed by stimulation with 2 ng/ml TGFβ1 or in combination with TGFβR1 inhibitor SB-431542 (System Bioscience, #ZRD-SB-02). At the indicated time points, cells were lysed in 500 µl whole-cell lysis buffer (1% NP40, 150 mM NaCl, 50 mM Tris-HCl pH 7.4, 2.5 mM NaF, 1 mM EDTA, 0.5 mM Na_3_VO_4_, 1 mg/ml deoxycholic acid, 2 μg/ml aprotinin and 200 μg/ml AEBSF). Lysates were kept on ice for 10 min, sonicated for 25 sec (Bandelin Sonopuls) and centrifuged for 10 min at 21,000 *g* at 4 °C. Protein concentration was determined by BCA assay (Pierce, #23225). For immunoprecipitation (IP), 700 µg of the protein was mixed with 2.5 µl of antibodies against Smad2/3 (BD Biosciences, #610842) and 25 µl 50% ProteinA sepharose-slurry (GE Healthcare, #17-0963-03). IP samples were rotated overnight at 4 °C, washed twice with whole-cell lysis buffer, once with TNE buffer (10 mM Tris, 100 mM NaCl, 1 mM EDTA and 100 µM Na_3_VO_4_) and boiled for 5 min at 95 °C. Samples were separated on 10% SDS-PAGE, transferred to nitrocellulose membrane (Amersham, #1060001), blocked for 1 h with 33% (v/v) Odyssey blocking buffer (LI-COR, #927-40010) in PBS and incubated with anti-Smad2/3 (BD Biosciences, #610842, 1:2,000), anti-pSmad3 (Cell Signaling Technology, #9520, 1:2,000) antibodies. Secondary antibodies coupled to IRDye infrared dyes (LI-COR, #926-32211 and #926-68070, 1:15,000) were used for detection with infrared Odyssey imager (LI-COR). Primary and secondary antibodies were diluted in 33% (v/v) Odyssey blocking buffer (LI-COR, #927-40010) in 0.2% TBS-Tween 20. Infrared Odyssey imager (LI-COR) was used for detection. Signal quantification was performed using the ImageQuant TL software (GE Healthcare). Independent replicates were scaled and averaged using methods previously described^38^.

### 2D migration assay

25,000 cells/well were seeded in 24-well plate (Zell Kontakt # 3231-20), allowed to attach overnight, growth factor-depleted for 3 h, stained with Hoechst (Santa Cruz, #sc-396575), stimulated with 2 ng/ml TGFβ1 alone or in combination with TGFβR1 inhibitor SB-431542 and imaged on an environment-controlled microscope (IX81, Olympus). Nine positions per well (3 × 3 grid) were acquired in 20 min intervals for 60 h. Images were stitched with the Grid/Collection ImageJ plugin. Single cell tracking was performed with the ImageJ Mtrack2 plugin.

### 3D collagen invasion assay

3D collagen gels were prepared as described previously^39^. Briefly, ice-cold 1 M HEPES buffer, 0.7 M NaOH, 10× PBS pH 8.0 and bovine skin collagen G solution (L1613, Biochrome) were mixed in 1:1:2:16 ratio. 50 µl of the resulting solution was added per well of a 96-well plate with a flat bottom (BD #353376). Plates were kept overnight at 4 °C and for 2 h at 37 °C to allow gelation of the collagen. After gelation, 12,500 cells per well were seeded on top of the matrix, allowed to adhere overnight, stimulated with 2 ng/ml TGFβ1 in FCS-depleted medium and allowed to invade for 4 days. Afterwards, cells were fixed in 3.7% paraformaldehide for 1 h and subsequently stained with Hoechst (Santa Cruz, #sc-396575). Collagen embedded cells were imaged using a LSM710 confocal microscope (Carl Zeiss) equipped with a EC Plan-Neofluar DIC 10 × /0.3 NA objective lens (Carl Zeiss). For each well, 2 × 2 tile z-stacks were acquired. Image analysis was performed using Imaris software (Bitplane). Spot detection algorithm was applied to assign coordinates for the center of each nucleus. Coordinates of all detected nuclei were exported and corrected by a conical plane function using the InvasionCorrection R package^40^. The threshold for invasion was set to 30 μm. Percentage of invaded cells and the invasion depth were the output.

### Cell viability and caspase activity assays

10,000 cells per well were seeded in 96-well plates, allowed to attach overnight and stimulated with 2 ng/ml TGFβ1 in growth factor-depleted medium or left untreated. Three days later the medium was replaced with culture DMEM, with or without TGFβ, and supplemented with different concentrations of cisplatin (Teva). CellTiter-Blue Viability (Promega) and Apo-ONE Homogeneous Caspase-3/7 (Promega) assays were used to measure the viability of cells and caspase 3/7 activity.

### RNA-Seq

SK-MES1 cells were seeded on 6-cm dishes at a density of 10^6^ cells/dish. Two days later, cells were growth factor-depleted for 3 hours and stimulated with 2 ng/ml TGFβ1 or left untreated. Total RNA was extracted at the indicated time points and sequenced using HiSeq 4000 (Illumina). The RNA sequencing results were aligned to the GRCh38 reference genome (Ensembl Release 79) and transcript quantification was performed using kallisto (v0.43.0)^41^. Subsequent transcript abundances were summarized to the gene level for analysis using tximport (v1.2.0)^42^. All ID mapping was performed using biomaRt (v2.30.0)^43^ and genes with read counts <10 were filtered out. Differential expression analysis was performed according to limma voom (v3.30.13)^44^ to obtain *F*-values and adjusted *P*-values. Adjusted *P*-values were calculated by multiple testing correction according to the Benjamini–Hochberg procedure. Gene set enrichment analysis (GSEA) was performed on the *F*-values of the differential expression analysis according to GAGE^45^. Upregulated GO cellular components terms (adjusted *P-*value < 0.01) were used for network analysis and visualized using REVIGO^11^ with allowed similarity of 0.5 and Cytoscape^46^.

### siRNA transfections

A pool of four targeting siRNAs (5 nM; SMARTpool, Dharmacon) was in-solution transfected during cell seeding using Lipofectamine RNAiMAX (ThermoFisher Scientific, #13778150) according to the manufacturer’s instructions. A pool of four non-targeting siRNA was used as a negative control (5 nM, Dharmacon, #D-001810-10-20). Experiments were started 36 hours after transfection. Sequences of used siRNAs are listed in the Supplementary Table S2.

### Tissue sample collection, characterization and preparation

Tissue samples were provided by Lung Biobank Heidelberg, a member of the accredited Tissue Bank of the National Center for Tumor Diseases (NCT) Heidelberg, the BioMaterialBank Heidelberg and the Biobank platform of the German Center for Lung Research (DZL). All patients provided written informed consent for the use of the tissue for research purpose. The study was approved by the local ethics committee of the University of Heidelberg (No. 270/2001). All research was performed in accordance with relevant guidelines/regulations. Tumor and matched distant (>5 cm) normal lung tissue samples from NSCLC patients (n = 362) who underwent resection for primary lung cancer at the Thoraxklinik at University Hospital, Heidelberg, Germany were collected. All diagnoses were made according to the 2004 WHO classification for lung cancer^47^ by at least two experienced pathologists. Tissues were snap-frozen within 30 minutes after resection and stored at −80 °C until the time of analysis. Only samples with a viable tumor content of ≥50% were used for subsequent analyses. For nucleic acid isolation 10–15 tumor cryosections (10–15 µm each) were prepared for each patient. The first and the last sections in each series were stained with hematoxylin and eosin (H&E) and were reviewed by an experienced lung pathologist to determine the proportions of viable tumor cells, stromal cells, normal lung cell cells, infiltrating lymphocytes and necrotic areas^48^.

### Total RNA isolation and cDNA synthesis

Frozen tumor cryosections were homogenized with the TissueLyser mixer-mill disruptor (Qiagen). Matched normal lung tissue pieces were homogenized using a Miccra D-8 rotor-stator homogenizer (Art-moderne Labortechnik). Total RNA was isolated from tissue or from cultured cells using an RNeasy Mini Kit (Qiagen). The quantity of RNA was measured with a NanoDrop ND-1000 Spectrophotometer (NanoDrop Technologies). The quality of total RNA was assessed with an Agilent 2100 Bioanalyzer and Agilent RNA 6000 Nano Kit (Agilent Technologies). Total RNA was transcribed to sscDNA with a Transcriptor First Strand cDNA Synthesis Kit (Roche) in three independent reactions that were afterwards pooled, mixed and stored at −80 °C until further analyses.

### Quantitative Real-Time PCR

qRT-PCR was performed using a LightCycler 480 (Roche) under MIQE guidelines^49^. CT values were calculated using the second derivative maximum method (Roche). To evaluate differences in patient gene expression, a relative quantification method based on the ΔΔCT method was performed^50^. Target genes were normalized with the geometric mean of two housekeeping genes (*ESD* and *RPS18*) for patient samples or four genes (*GUSB, GAPDH, G6PD and HPRT*) for SK-MES1 samples. Sequences of used PCR primers are listed in the Supplementary Table S3.

### Statistical analyses

The qRT-PCR data were statistically analyzed under REMARK criteria^51^ with SPSS 22.0 for Windows (IBM). The endpoint of the study was overall survival. Survival was calculated from the date of surgery until the last date of contact or death. Univariate analysis of survival data was performed according to Kaplan and Meier^52^ and using Cox proportional hazard models. Multivariate survival analysis was performed using the Cox proportional hazards model. The non-parametric Mann-Whitney U test was used to investigate significant differences between the patient groups.

### Data availability

The RNA-Seq data was deposited at the Sequence Read Archive (SRA) and is accessible via the Gene Expression Omnibus ID GSE95536.

## Electronic supplementary material

Supplementary Information
