# Supplementary material for: Expression ratio of the TGFβ-inducible gene MYO10 is prognostic for overall survival of squamous cell lung cancer patients and predicts chemotherapy response
Source: Sci Rep. 2018 Jun 22;8:9517. doi: 10.1038/s41598-018-27912-1 (PMC6015003; doi:10.1038/s41598-018-27912-1)
Supplement: Supplementary file 1 — Supplementary Information [file 41598_2018_27912_MOESM1_ESM.pdf]

Expression ratio of the TGFβ-inducible gene *MYO10* is prognostic for overall survival of squamous cell lung cancer patients and predicts chemotherapy response

Dvornikov D[1,2,3*], Schneider MA[2,4*], Ohse S[5*], Szczygieł M[1,2,3], Titkova I[1,3], Rosenblatt M[6], Muley T[2,4], Warth A[2,7], Herth FJ[2,8], Dienemann H[2,9], Thomas M[2,10], Timmer J[6,11,12], Schilling M[1], Busch H[5,13†], Boerries M[5,14†], Meister M[2,4†], and Klingmüller U[1,2†]

1: Division Systems Biology of Signal Transduction, German Cancer Research Center (DKFZ), 69120 Heidelberg, Germany.

2: Translational Lung Research Center Heidelberg (TLRC), German Center for Lung Research (DZL), 69120 Heidelberg, Germany.

3: Faculty of Biosciences, Heidelberg University, 69120 Heidelberg, Germany.

4: Translational Research Unit, Thoraxklinik at University Hospital Heidelberg, 69126 Heidelberg, Germany.

5: Institute of Molecular Medicine and Cell Research, University of Freiburg, 79104 Freiburg, Germany.

6: Institute of Physics, University of Freiburg, 79104 Freiburg, Germany.

7: Institute of Pathology, Heidelberg University, 69120 Heidelberg, Germany.

8: Department of Pneumology and Critical Care Medicine, Thoraxklinik at University Hospital Heidelberg, 69126 Heidelberg, Germany.

9: Department of Surgery, Thoraxklinik at University Hospital Heidelberg, 69126 Heidelberg, Germany.

10: Department of Thoracic Oncology, Thoraxklinik at University Hospital Heidelberg, 69126 Heidelberg, Germany.

11: Freiburg Centre for Systems Biology, University of Freiburg, 79104 Freiburg, Germany.

12: BIOSS Centre for Biological Signaling Studies, University of Freiburg, 79104 Freiburg, Germany.

13: Institute of Experimental Dermatology & Institute of Cardiogenetics, University of Lübeck, 23562 Lübeck, Germany.

14: German Cancer Consortium (DKTK), German Cancer Research Center (DKFZ), 69120 Heidelberg, Germany.

Correspondence: Prof. Dr. Ursula Klingmüller, Division Systems Biology of Signal Transduction, German Cancer Research Center (DKFZ), Im Neuenheimer Feld 280, 69120 Heidelberg, Germany

E-mail: u.klingmueller@dkfz.de
Tel +49 6221 424481
Fax +49 6221 422288

*These authors contributed equally to this work.

†Shared senior authorship.

Supplementary Information

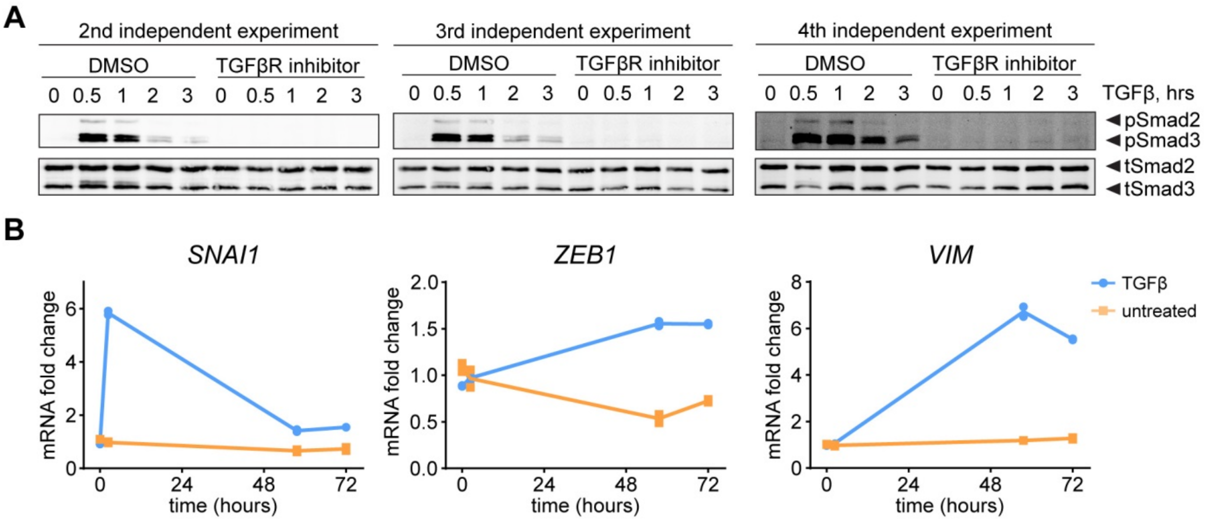

Supplementary Figure S1. **TGFβ treatment triggers Smad phosphorylation and EMT gene expression in SK-MES1 cells**. (**A**) Additional independent experiments of Smad2/3 activation in SK-MES1 cells upon TGFβ treatment. Cells were pretreated with SB-431542 or DMSO and then stimulated with 2 ng/ml TGFβ1. Full-length blots are shown in Supplementary Fig S6. (**B**) Additional independent experiments for upregulation of EMT genes in SK-MES1 cells upon stimulation with TGFβ. Growth factor-depleted SK-MES1 cells were stimulated with 2 ng/ml TGFβ1 or left untreated. RNA was extracted and analyzed using qRT-PCR. mRNA expression was normalized to four housekeepers: *GUSB*, *HPRT*, *GAPDH* and *G6PD*. Each dot represents a biological replicate.

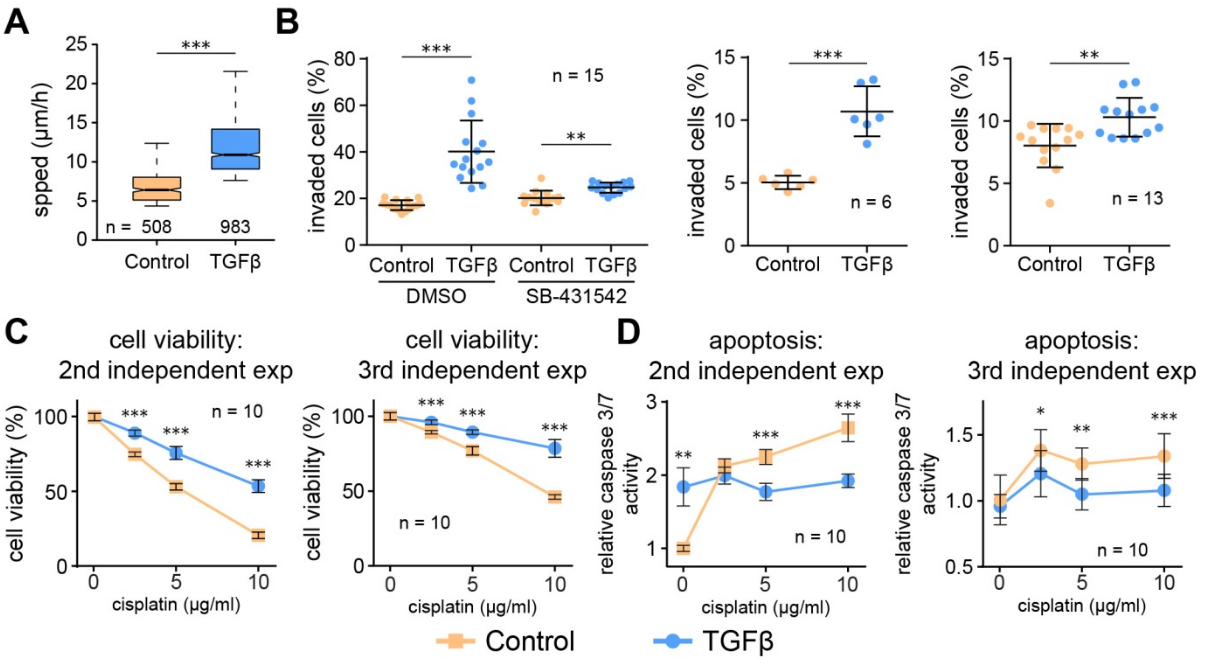

Supplementary Figure S2. **TGFβ treatment increases cancer cell invasion and cisplatin resistance of squamous lung carcinoma cells SK-MES1**. (**A**) Additional independent experiment of 2D migration. SK-MES1 cells were seeded in 24-well plate, stimulated with 2 ng/ml TGFβ1 and imaged for 60 hours. Migration speed from each single cell track was quantified. Center lines show the medians; box limits indicate the 25th and 75th percentiles; whiskers extend to 5th and 95th percentiles; n indicates number of quantified single cell tracks per condition. Statistical analysis was performed using one-way ANOVA; ***$P < 0.001$. (**B**) Additional independent experiments of collagen 3D invasion assay. SK-MES1 cells were seeded in 96-well plate with precast collagen gels and allowed to attach overnight, serum-starved for three hours, pretreated with either SB-431542 or DMSO, stimulated with 2 ng/ml TGFβ1, allowed to invade for four days, stained with Hoechst and imaged with a confocal microscope. Amount of invaded cells into the collagen gel and invasion depth were assessed. Every dot corresponds to a biological replicate (n). Data are presented as median and SD. Statistical analysis was performed using one-way ANOVA; **$P < 0.01$; ***$P < 0.001$. (**C, D**) Additional independent experiments of cell viability and caspase 3/7 activity assays. SK-MES1 cells were seeded in 96-well plate, stimulated with either 2 ng/ml TGFβ1 or left untreated for 3 days and then exposed to increasing doses of cisplatin for 3 days. Cell viability and caspase 3/7 activity was assessed. Data presented correspond to mean and SD, n is the number of biological replicates. Statistical analysis was performed using one-way ANOVA; *$P < 0.05$; **$P < 0.01$; ***$P < 0.001$.

A

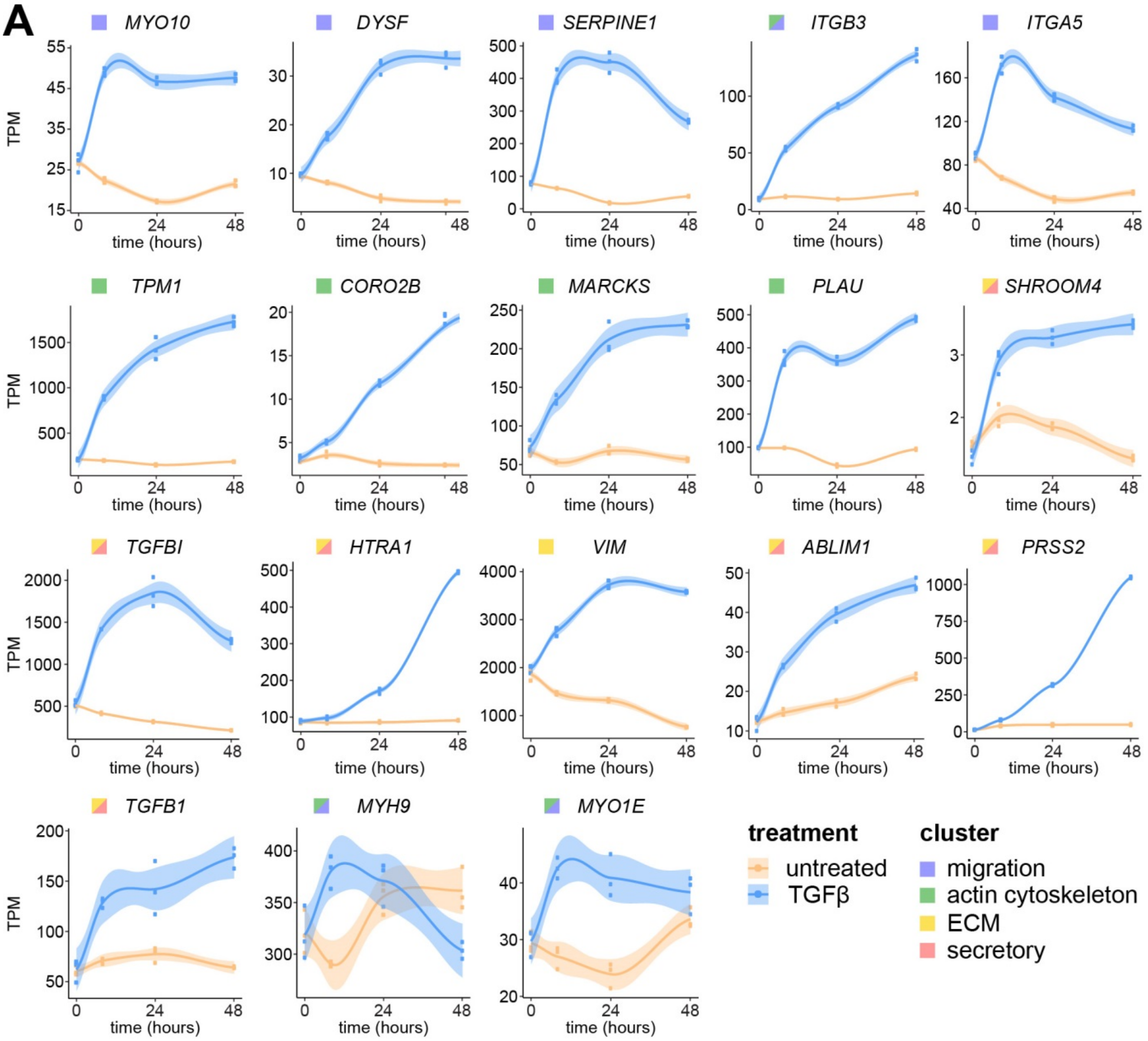

B

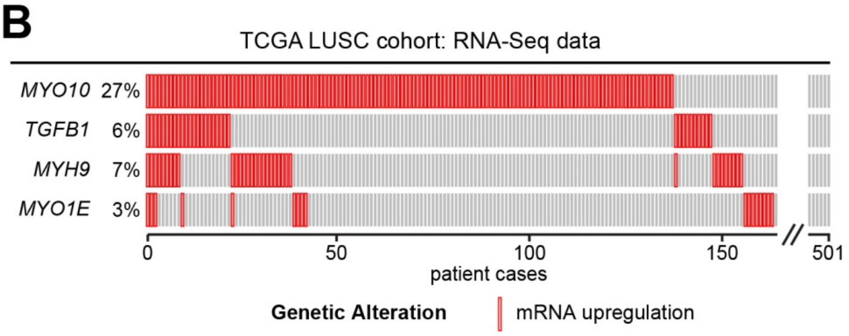

Tendency towards co-occurrence

gene A	gene B	*P*-value
MYO10	*MYH9*	7.64×10^{-8}
MYO10	*TGFB1*	5.12×10^{-7}
TGFB1	*MYH9*	1.22×10^{-5}
TGFB1	*MYO1E*	1.81×10^{-2}
MYO10	*MYO1E*	2.05×10^{-2}
MYH9	*MYO1E*	2.24×10^{-2}

Supplementary Figure S3. Time-resolved dynamics of candidate genes. (**A**) Cells were growth factor-depleted for three hours and stimulated with 2 ng/ml TGFβ1 or left untreated. mRNA was extracted and sequenced using HiSeq 4000. Data are presented in TPM (transcripts per million) values. Each dot represents a biological replicate; shaded areas correspond to standard error. (**B**) TCGA RNA-Seq expression data of candidate myosin and *TGFB1* genes in LUSC. The significance of the association was tested using Fisher's exact test.

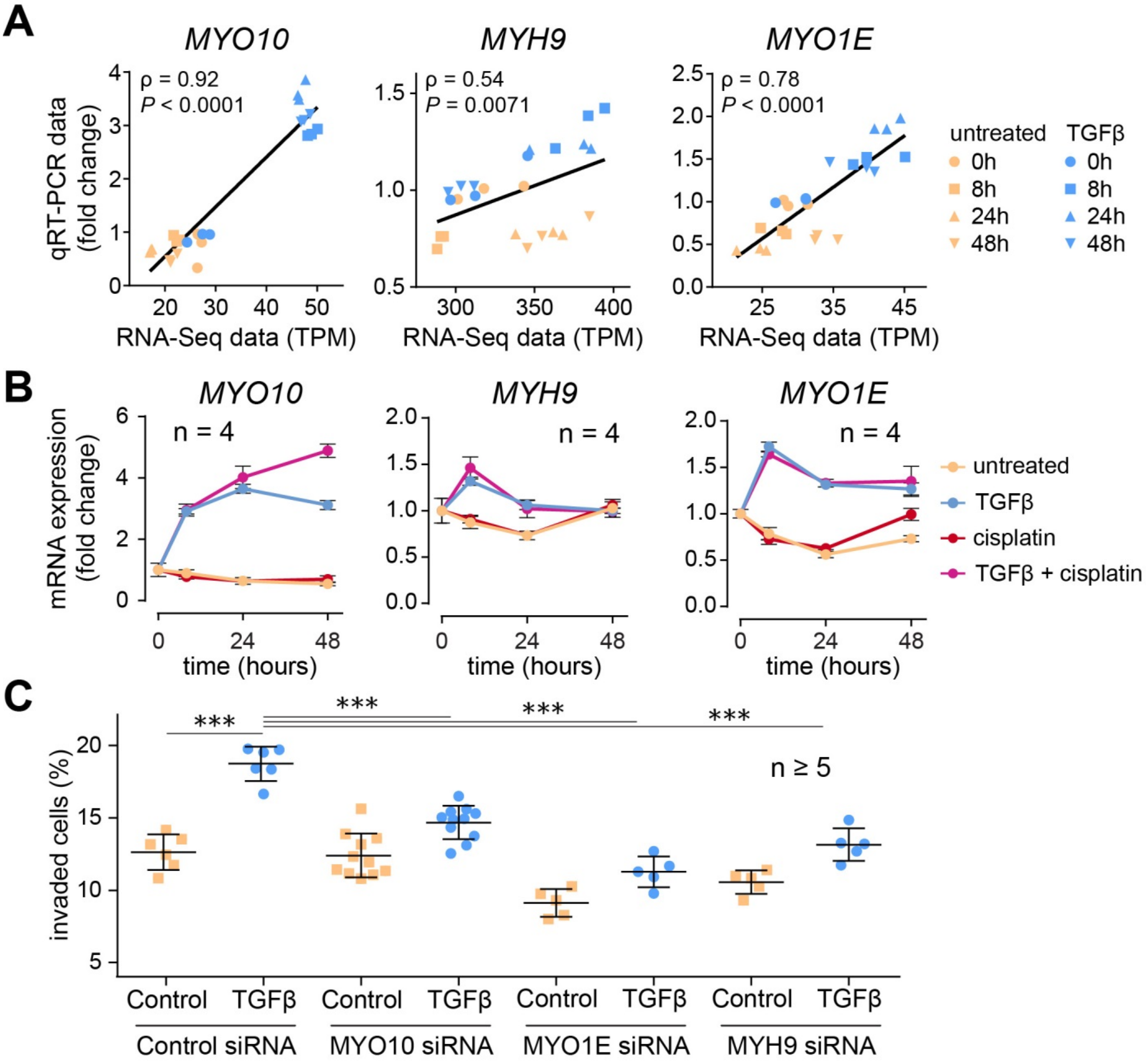

Supplementary Figure S4. Knockdown of TGFβ-inducible myosins abrogates TGFβ-mediated cancer cell invasion. (**A**) Comparison of *MYO10*, *MYH9* and *MYO1E* mRNA expression data assessed by RNA-Seq and qRT-PCR methods. Spearman correlation coefficient and *P*-value determined by two-tailed *t*-test are displayed. (**B**) Expression of *MYO10*, *MYH9* and *MYO1E* mRNA in response to cisplatin treatment alone or in combination with 2 ng/ml TGFβ. Data presented correspond to mean and SD from four biological replicates. (**C**) Additional independent experiment of collagen 3D invasion assay. SK-MES1 cells were transfected with siRNAs for 36 hours and stimulated with 2 ng/ml TGFβ for four days. Amount of invaded cells into the collagen gel was assessed. Every dot corresponds to a biological replicate (n). Data are presented as median and SD. Statistical analysis was performed using one-way ANOVA; ***P < 0.001.

A

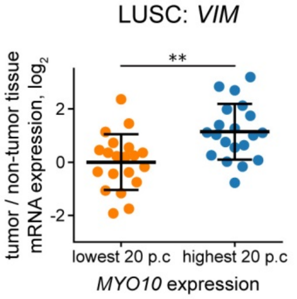

B

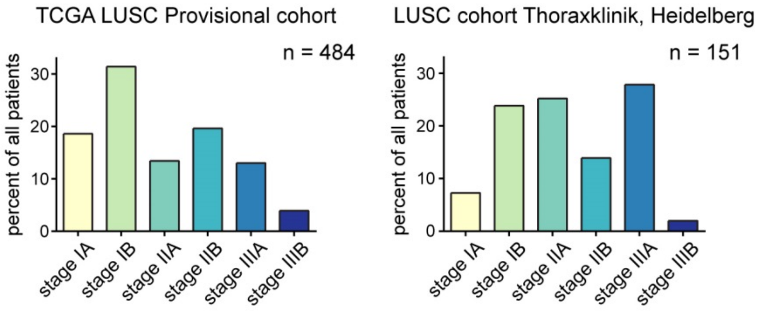

C

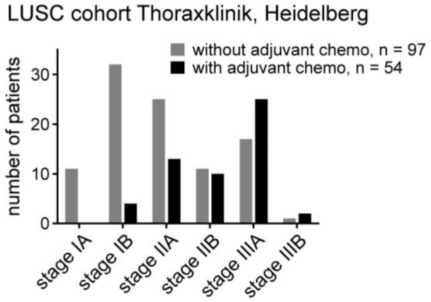

D

Survival	Population	Number of patients	Median survival (months)	95 % CI
Overall survival	**SQCC without ChT**	**97**	**32.90**	**28.4–37.4**
	death	38	26.3	19.5–33.1
	alive	59	37.1	31.4–42.9
Overall survival	**SQCC with ChT**	**54**	**39.7**	**33.8–45.6**
	death	21	20.8	15.6–25.9
	alive	33	51.8	45.6–58.0
Disease-free survival	**SQCC without ChT**	**97**	**28.4**	**23.9–33.0**
	death	49	20.5	14.6–26.4
	alive	48	36.5	30.1–42.8
Disease-free survival	**SQCC with ChT**	**54**	**35.6**	**29.5–41.6**
	death	25	19.5	13.0–26.0
	alive	29	49.4	43.0–56.0

Supplementary Figure S5. Patients with higher *MYO10* mRNA expression ratio have higher expression of EMT marker genes. (**A**) Difference of *VIM* expression depending on *MYO10* mRNA expression ratio was tested by unpaired *t*-test; $^{**}P < 0.01$. 20 patient cases (p.c) with lowest *MYO10* ratio were compared to 20 patient cases with highest *MYO10* ratio. (**B**) Comparison of tumor stage distrubition in TCGA and our LUSC cohorts. (**C**) Tumor stage distribution of LUSC patients who either received (n=54) or did not receive (n=97) adjuvant chemotherapy after resection of the primary tumor. (**D**) Comparison of overall survival and disease-free survival of LUSC patients who either received (n=54) or did not receive (n=97) adjuvant chemotherapy after resection of the primary tumor.

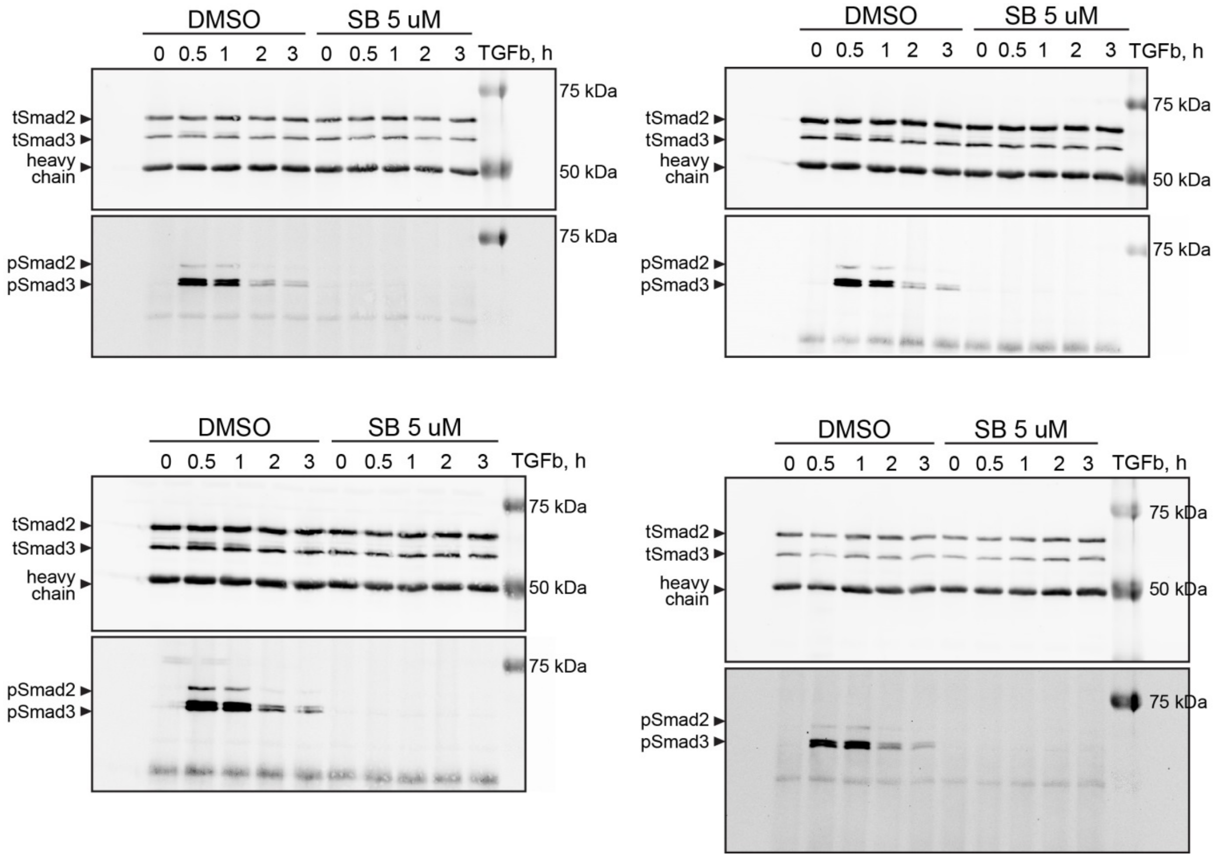

Supplementary Figure S6. Full-length immunoblots for Figures 1A and S1A.

Supplementary Table S1. Patient clinical and pathological characteristics

Parameter	all patients, n = 362		squamous cell carcinoma patients, n = 151	
	**n**	**(%)**	**n**	**(%)**
Median Age	65 (38-88)		65 (42-83)	
Gender	**362**		**151**	
Male	250	69	125	83
Female	112	31	26	17
Histology				
LUAD	211	58		
LUSC	151	42		
Therapy				
OP	212	59	90	60
OP/RT	13	4	7	5
OP/CT	100	28	45	30
OP/RT/CT	37	10	9	6
P stage				
IA	37	10	11	7
IB	90	25	36	24
IIA	70	19	38	25
IIB	51	14	21	14
IIIA	105	29	42	28
IIIB	9	2	3	2
ECOG				
0	320	88	134	89
1	32	9	11	7
2	4	1	2	1
n.d.	8	2	4	3
smoking status				
Non-smoker	40	11	1	0
Ex-smoker	189	52	84	56
current smoker	131	36	66	44
n.d.	2	0		

OP = surgery, CT = chemotherapy, RT = radiotherapy, ECOG = Eastern Cooperative Oncology Group, n.d. = no data

Supplementary Table S2: siRNA sequences

target	art #	sequence
MYO10	J-007217-05	CGACAUAAAUCUCAACUUG
MYO10	J-007217-06	CGUCGUAGCUGAUGUCUUA
MYO10	J-007217-07	GCGGGAGAAUUGUAGAUUA
MYO10	J-007217-08	GGAGGAAAUUUCAGGGAAU
MYH9	J-007668-05	GUAUCAAUGUGACCGAUUU
MYH9	J-007668-06	CAAAGGAGCCCUGGCGUUA
MYH9	J-007668-07	GGAGGAACGCCGAGCAGUA
MYH9	J-007668-08	CGAAGCGGGUGAAAGCAAA
MYO1E	J-019919-09	GUACUUAAUCGGACGAGAA
MYO1E	J-019919-10	GUUCAAGGGUGUAAAGCGA
MYO1E	J-019919-11	GGGUAAAGCAUCAAGUCGA
MYO1E	J-019919-12	UAAAGGAAUCGGCGAACAA

Supplementary Table S3: qRT-PCR primers

Gene name	NCBI Entry	Forward Primer	Reverse Primer	UPL Probe
MYO10	NM_012334.2	cccagcagctgattcaagat	cgggttccgcttgtaaatc	31
MYH9	NM_002473.5	tggaggaccagaactgcaa	ggttggtggtgaactcagcta	11
MYO1E	NM_004998.3	gtgtcacagacgccagagag	gccgactggttgtttgtctc	24
TWIST1	NM_000474.3	agctacgccttctcggtct	ccttctctggaaacaatgacatc	58
VIM	NM_003380.3	tacaggaagctgctggaagg	accagagggagtgaatccag	13
SNAI2	NM_003068.4	tggttgcttcaaggacacat	gcaaatgctctgttgcagtg	7
ESD	NM_001984.1	tcagtctgcttcagaacatgg	cctttaatattgcagccacga	50
RPS18	NM_022551.2	cttccacaggaggcctacac	cgcaaaatatgctggaacttt	46
GUSB	NM_000181.3	cgccctgcctatctgtattc	tccccacagggagtgtgtag	57
GAPDH	NM_002046	agccacatcgctcagacac	gcccaatacgaccaaatcc	60
G6PD	NM_000402	ctgcagatgctgtgtctggt	tgcatttcaacaccttgacc	22
HPRT	NM_000194.2	tgaccttgatttattttgcatacc	cgagcaagacgttcagtcct	73
